# Thrombotic thrombocytopenic purpura and macrophage activation syndrome secondary to Sjögren’s syndrome: a case report

**DOI:** 10.3389/fimmu.2026.1810384

**Published:** 2026-07-03

**Authors:** Xiaoya Liu, Maiqi Liu, Mengjiao Yao, Haohu Wu, Lin Bian, Xiuhua Wang, Tingting Zhai, Qing Wang, Yanfeng Hou

**Affiliations:** 1 The First Affiliated Hospital of Shandong First Medical University and Shandong Provincial Qianfoshan Hospital, School of Clinical Medicine, Shandong First Medical University, Jinan, Shandong, China; 2 Department of Rheumatology and Autoimmunology, Shandong Province University Clinical Immunology Translational Medicine Laboratory, The First Affiliated Hospital of Shandong First Medical University and Shandong Provincial Qianfoshan Hospital, Jinan, Shandong, China

**Keywords:** macrophage activation syndrome, plonmarlimab, ruxolitinib, Sjögren’s syndrome, thrombotic thrombocytopenic purpura

## Abstract

Sjögren’s syndrome (SS) is a systemic autoimmune disorder frequently associated with diverse hematological manifestations. Immune-mediated thrombotic thrombocytopenic purpura (iTTP) and macrophage activation syndrome (MAS) represent rare, life-threatening complications, yet the consecutive occurrence of both conditions in the same SS patient is exceedingly rare. We report a 20-year-old female diagnosed with SS following drug-induced liver injury. In July 2023, she developed acute iTTP (0% ADAMTS13 activity, with positive inhibitory antibodies) shortly after the initiation of immunomodulatory therapy, which responded to plasma exchange and corticosteroids. In December 2023, following *Mycoplasma pneumoniae* infection, she presented with MAS, fulfilling seven of eight hemophagocytic lymphohistiocytosis (HLH)-2004 criteria. Management included high-dose methylprednisolone, etoposide, and ruxolitinib, followed by plonmarlimab (anti-granulocyte-macrophage colony-stimulating factor (GM-CSF) antibody) under a clinical trial protocol. This unique case underscores the complexity of SS-associated hematological emergencies. Based on our findings, we propose a dual-pathway hypothesis for these complications: firstly, elevated IL-6 may facilitate the thrombotic cascade in SS-associated iTTP; secondly, the interferons-Janus kinases-signal transducer and activator of transcription (IFN-JAK-STAT) pathway’s aberrant activation likely creates a mechanistic intersection that predisposes SS patients to MAS. While the efficacy of targeted agents like tocilizumab, ruxolitinib, and plonmarlimab appears promising in this context, these therapeutic avenues strictly warrant further systematic investigation through larger clinical cohorts.

## 1 Introduction

Sjögren’s syndrome (SS) is a chronic autoimmune disease frequently associated with complex extraglandular manifestations, including severe hematological complications (1). Immune-mediated thrombotic thrombocytopenic purpura (iTTP) and macrophage activation syndrome (MAS) are individually rare diseases, with annual incidences in the general population typically below 15 and 10 per million, respectively. iTTP is primarily driven by autoantibody-mediated ADAMTS13 deficiency, whereas MAS manifests as a hyperinflammatory cytokine storm (2–5). While recognized as life-threatening Sjögren’s syndrome (SS) complications, their consecutive manifestation in a single patient is exceptionally rare (6, 7).

The clinical management of such sequential crises presents formidable challenges, as their clinical signatures frequently overlap with and may critically mask the baseline activity of the underlying rheumatic disease. We report a highly unusual case of SS sequentially complicated by iTTP and MAS. By evaluating this unique clinical course—marked by successful intervention with the JAK inhibitor ruxolitinib and the novel anti-granulocyte-macrophage colony-stimulating factor (GM-CSF) antibody, plonmarlimab—we propose a dual-pathway hypothesis involving IL-6 signaling and the interferons-Janus kinases-signal transducer and activator of transcription (IFN-JAK-STAT) axis to elucidate the potential mechanistic intersections between these three distinct entities. This framework aims to provide a diagnostic and therapeutic roadmap for managing such refractory multi-system autoimmune complications.

## 2 Case presentation

**Figure 1:**
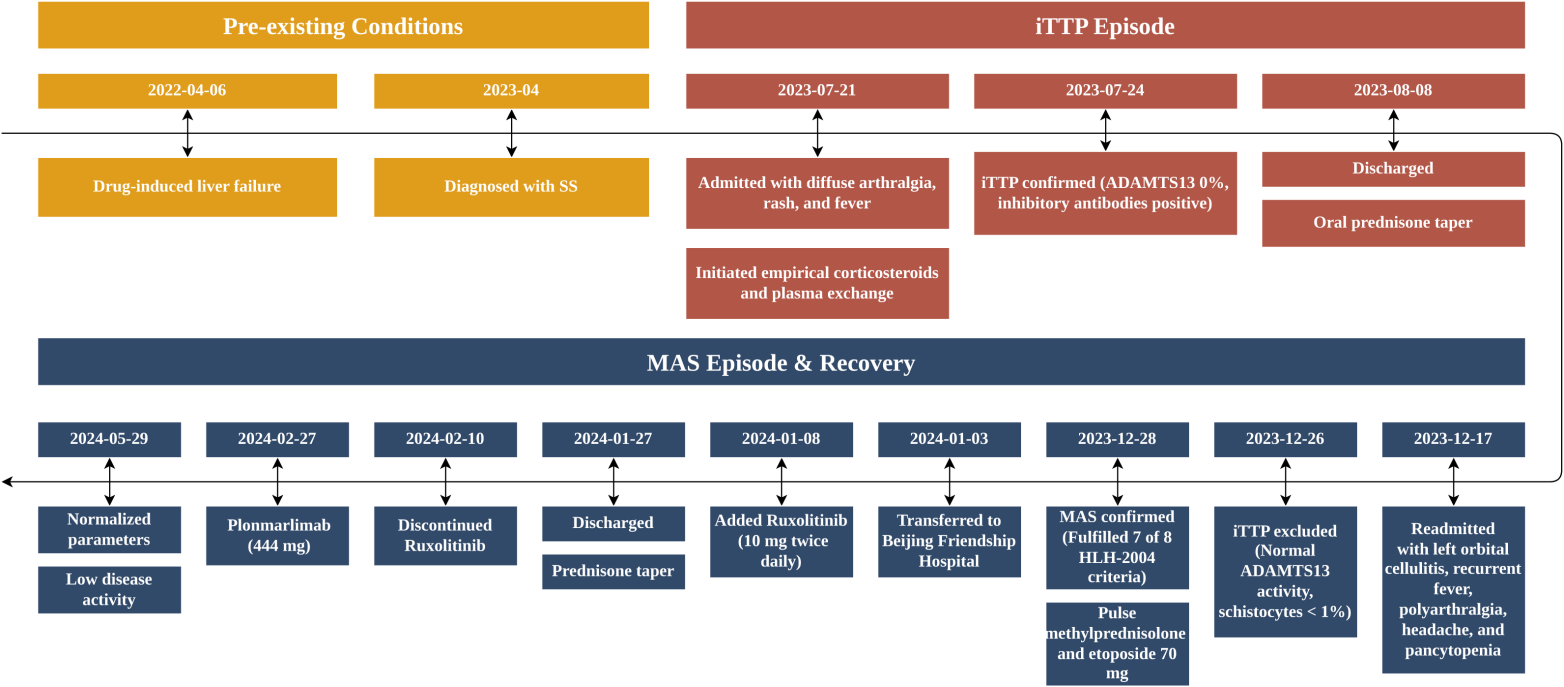
Timeline of the patient’s overall clinical course. The diagram details the pre-existing conditions (including drug-induced liver failure and Sjögren’s syndrome (SS) diagnosis), the episode of immune-mediated thrombotic thrombocytopenic purpura (iTTP), and the subsequent episode of macrophage activation syndrome (MAS) along with the recovery phase.

This is a single-patient case report conducted at a single tertiary center. The patient’s clinical course was documented between 2022 and 2024 (**Figure 1**).

### 2.1 Patient information

The patient, a 19-year-old female student, had no history of smoking or alcohol consumption, no family history of autoimmune or hematological disorders, no known drug allergies, and no history of psychiatric disorders. In April 2022, she developed acute drug−induced liver failure after using Chinese herbal medicine. Liver biopsy excluded autoimmune hepatitis, and she improved following artificial liver support therapy.

In April 2023, the patient complained of dry mouth and dry eyes. Examination revealed positive anti-SSA and anti-AMA-M2, alkaline phosphatase 113 U/L, gamma-glutamyl transferase 41 U/L, alanine aminotransferase 34 U/L, Schirmer’s test ≤5 mm/5 min, and tear break-up time <5 s. With primary biliary cholangitis excluded given the normal liver enzymes and the absence of cholestatic symptoms, she was thus diagnosed with SS based on fulfillment of the 2016 American College of Rheumatology (ACR) and European Alliance of Associations for Rheumatology (EULAR) criteria (8). Treatment consisted of 12 mg of oral methylprednisolone administered once daily.

### 2.2 Immune-mediated thrombotic thrombocytopenic purpura

On July 21, 2023, within 48 hours of initiating hydroxychloroquine (HCQ 400 mg daily) and total glucosides of paeony (TGP 1,800 mg daily), she experienced diffuse arthralgia, a generalized maculopapular rash, and a fever of 40 °C. Upon admission, she exhibited multisystem dysfunction: white blood cells: 10.79 × 10^9^/L, platelets (PLT): 8 × 10^9^/L with schistocytes present at ≥2 per high-power field, lactate dehydrogenase (LDH) peak: 2,723 U/L, indirect bilirubin: 29.1 μmol/L, urine occult blood: +++, Coombs test-negative, ferritin >40,000 ng/mL, creatinine: 117 μmol/L, interleukin-6 (IL-6): 9.80 pg/mL (reference range: 0–5.4 pg/mL), and interleukin-10 (IL-10): 13.79 pg/mL (reference range: 0–12.9 pg/mL), blood cultures: negative, and viral screening: negative.

**Figure 2:**
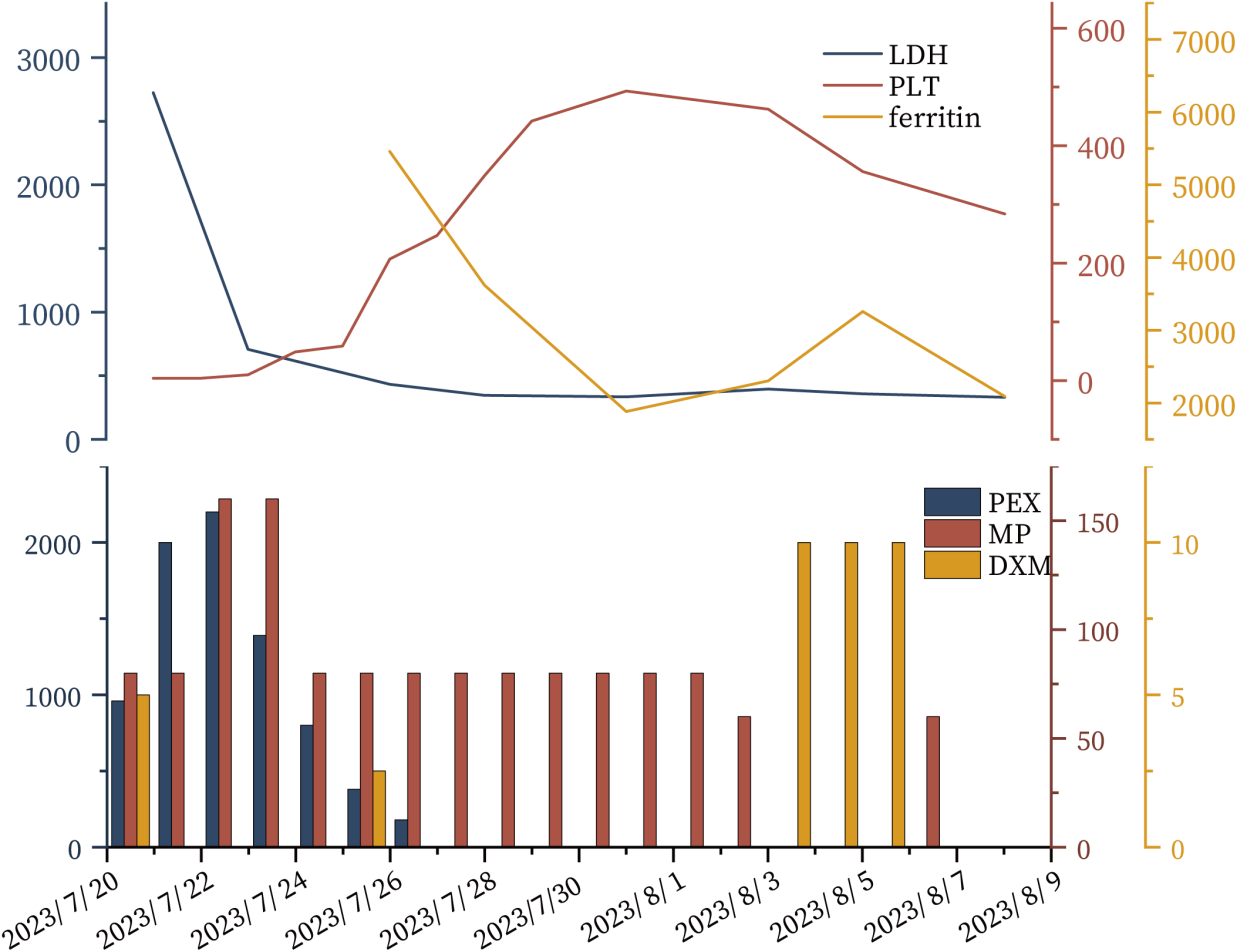
The patient’s clinical course of thrombotic thrombocytopenic purpura. Platelets (PLT, × 10^9^/L), serum ferritin (ferritin, ng/mL), lactate dehydrogenase (LDH, U/L), dexamethasone (DXM, mg), methylprednisolone (MP, mg), and plasma exchange (PEX, mL).

A multidisciplinary team review considered a diagnosis of iTTP, supported by the PLASMIC score (9). Empirical corticosteroids and plasma exchange therapy were started (**Figure 2**). iTTP was confirmed on July 24 with 0% ADAMTS13 activity and inhibitory antibodies. The fever had subsided and the rash was fading by July 27, 2023. However, after starting mosapride on August 4, 2023, the patient developed an allergic reaction. By August 8, 2023, her laboratory parameters had significantly improved: white blood cells: 13.4 × 10^9^/L, platelets: 284 × 10^9^/L, LDH: 331 U/L, ferritin: 2,089.75 ng/mL, triglycerides: 1.63 mmol/L, IL-6: 3.34 pg/mL, and IL-10: 27.01 pg/mL. She was discharged on oral prednisone, tapering from 50 mg to 20 mg daily (5 mg reduction biweekly).

### 2.3 Macrophage activation syndrome

On October 30, 2023, she developed left orbital cellulitis, which was followed by polyarthralgia, headache, and a fever of 39.5 °C. Treatment with amoxicillin and ceftriaxone resulted in hypersensitivity reactions. She was readmitted on December 17, 2023.

**Figure 3:**
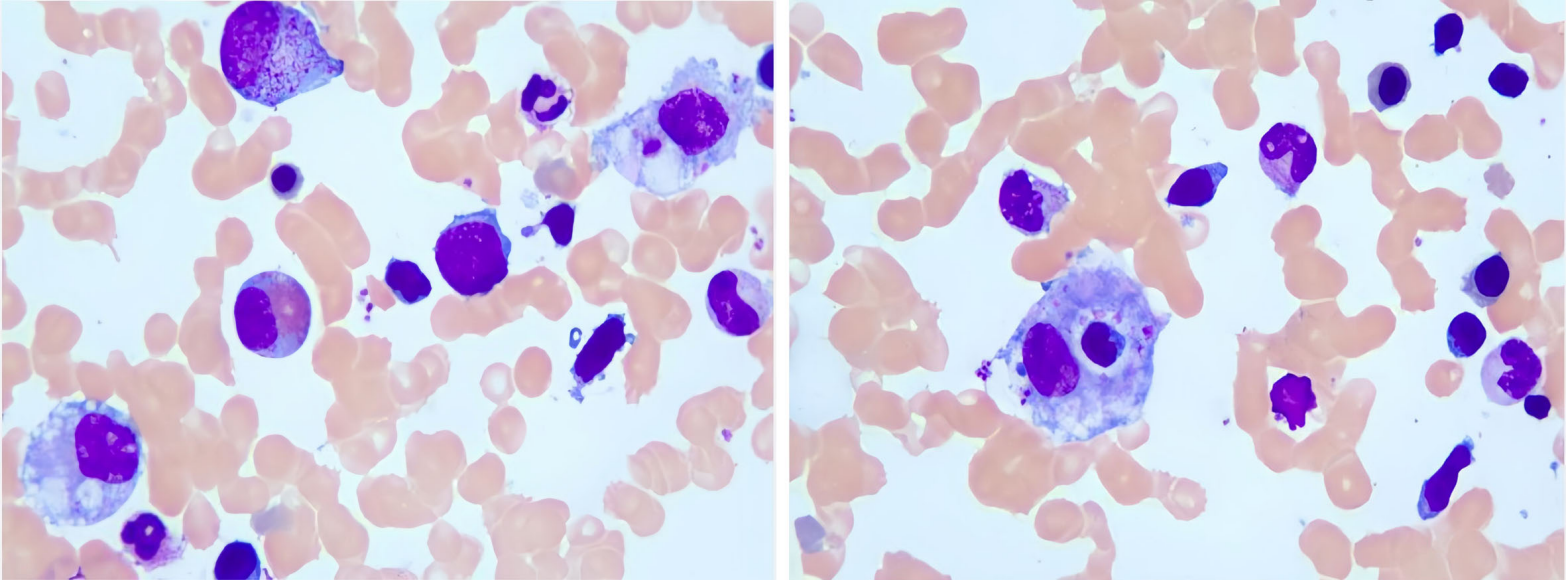
Bone marrow aspirate smear (Wright-Giemsa stain, 1000× magnification under oil immersion). The image demonstrates prominent hemophagocytosis, characterized by a large macrophage engulfing hematopoietic cells.

Admission laboratory findings: LDH: 1,196 U/L, aspartate aminotransferase/alanine aminotransferase: 1.04, PLT: 94 × 10^9^/L, international normalized ratio: 1.25, fibrinogen: 1.16 g/L, white blood cells: 13.42 × 10^9^/L, neutrophils: 11.51 × 10^9^/L, *Mycoplasma pneumoniae* positive, and Epstein-Barr virus negative. iTTP recurrence was highly suspected during multidisciplinary consultations, but sepsis and MAS could not be ruled out. However, ADAMTS13 activity was normal, and schistocytes were <1%. iTTP was then excluded. Additional laboratory tests revealed hypofibrinogenaemia (1.16 g/L), bone marrow hemophagocytosis (**Figure 3**), splenomegaly, and markedly elevated soluble CD25 (366.1 IU/mL; reference range: 17.1–56.6 IU/mL). No harmful mutations were found by whole-exome sequencing. By December 27, 2023, the patient had fulfilled seven of eight hemophagocytic lymphohistiocytosis (HLH)-2004 criteria (with NK cell activity remaining preserved at 39.0% as the sole unmet criterion), confirming the diagnosis of MAS in conjunction with the supportive diagnostic framework of the 2016 ACR and EULAR criteria (10, 11). Thus, methylprednisolone (1,000 mg for three days, then 500 mg for three days) and intravenous human immunoglobulin (20 g/d) were added to the regimen.

For further management, the patient was transferred to Beijing Friendship Hospital on January 3, 2024. A follow-up examination upon transfer showed: alanine aminotransferase: 260.30 U/L, aspartate aminotransferase: 137.60 U/L, LDH: 372.00 U/L, hemoglobin: 88.0 g/L, fibrinogen: 0.98 g/L, platelet count: 102 × 10^9^/L, and ferritin: 3,196.04 ng/mL. Methylprednisolone was continued at 80 mg/d for 5 days. The patient remained afebrile after admission; however, as ferritin levels stayed markedly elevated at 4,758.77 ng/mL on January 7, ruxolitinib (10 mg twice daily) was added to the regimen on January 8. The intravenous methylprednisolone dose was subsequently tapered (80 mg/d for 2 days, 60 mg/d for 7 days, and 40 mg/d for 3 days). On January 20, therapy was transitioned to oral methylprednisolone at 30 mg/d (15 mg twice daily). The patient was discharged on January 27, and oral prednisone was progressively tapered to 25 mg/d for 7 days, 20 mg/d for 7 days, and 18 mg/d for 7 days.

**Figure 4:**
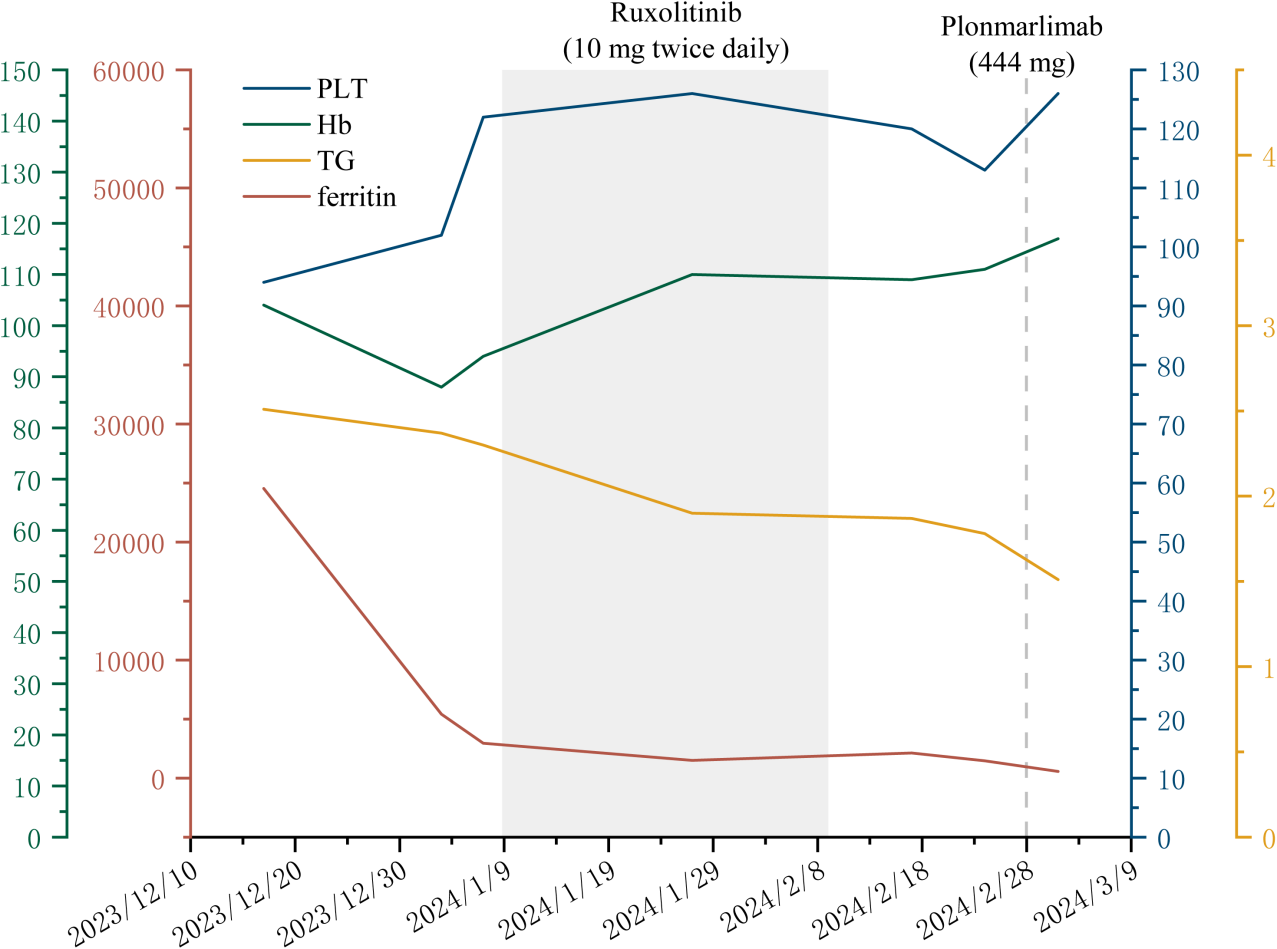
The patient’s clinical course of macrophage activation syndrome. Platelets (PLT, × 10^9^/L), hemoglobin (Hb, g/L), triglycerides (TG, mmol/L), serum ferritin (ferritin, ng/mL), ruxolitinib (10 mg twice daily), and plonmarlimab (444 mg).

Due to financial constraints, ruxolitinib was discontinued on February 10. A follow-up evaluation on February 17 indicated an incomplete remission of laboratory parameters. The oral prednisone dose was adjusted to 20 mg/d, and participation in a plonmarlimab (a novel anti-GM-CSF blocking antibody) clinical trial (trial registration number: NCT07034209, protocol ID: THZ0103-201) was recommended. After providing informed consent and meeting the inclusion criteria, baseline parameters were reassessed, and plonmarlimab was initiated on February 28 at a dose of 444 mg (10 mg/kg), with methylprednisolone maintained at 18 mg/d (**Figure 4**). Ultimately, the patient withdrew from the clinical trial for other reasons.

Methylprednisolone was maintained at 16 mg/d from March 15 to March 28, and subsequently tapered by 2 mg per week starting March 29, reaching a 4 mg/d maintenance dose in early May. A comprehensive follow-up on May 29 revealed normalized parameters: hemoglobin 127.0 g/L, platelet count 242 × 10^9^/L, C-reactive protein 3.2 mg/L, and ferritin 8.74 ng/mL. Liver function tests were unremarkable (alanine aminotransferase 37.10 U/L, aspartate aminotransferase 13.30 U/L, total bilirubin 6.40 µmol/L, direct bilirubin 3.20 µmol/L), and triglycerides were 1.39 mmol/L. Peripheral blood smears showed no schistocytes, ADAMTS13 activity remained normal, and complement C3, C4, as well as soluble CD25 levels had all returned to normal ranges. Clinically, the patient reported no fever, fatigue, or joint pain. As of her last documented clinical evaluation on May 29, 2024, there was no evidence of sequelae or clinical recurrence of TTP and MAS, and the patient’s Sjögren’s syndrome remains in a state of low disease activity.

The patient reported profound distress and exhaustion from the rapid onset of recurrent, life-threatening conditions. However, the multidisciplinary approach and diverse treatment strategies brought her immense psychological relief, ultimately resulting in her current relapse-free state and restored quality of life.

## 3 Discussion

SS is a chronic autoimmune disease characterized by lymphocytic infiltration and autoantibody deposition, which can include systemic multi-organ symptoms as well as hypofunction of the salivary and lacrimal glands (12). Serum autoantibodies are present in most patients with SS. Specific autoantibodies, such as anti-Ro/SSA, are strongly associated with a systemic disease phenotype, higher immunological activity, and hematological abnormalities, underscoring the need for close hematological monitoring in these patients (13, 14). Approximately 34–44% of individuals with SS have hematological involvement, which frequently manifests as cytopenias like anemia, leukopenia, thrombocytopenia, and pancytopenia (1, 15). Severe complications may include Evans syndrome, disseminated intravascular coagulation, iTTP, and MAS (6, 16). Notably, the clinical and laboratory features of these severe complications often overlap with general SS manifestations, leading to significant diagnostic challenges. The classic presentation of iTTP includes fever, thrombocytopenia, hemolytic anemia, renal impairment, and an ADAMTS13 activity below 10% (17). When accompanying coagulation abnormalities, including hypofibrinogenemia, and a marked elevation in D-dimer, disseminated intravascular coagulation should be considered. Evans syndrome usually concurs with autoimmune thrombocytopenia and direct Coombs test-positive autoimmune hemolysis (18). The manifestations of MAS are diverse and non-specific, including fever, splenomegaly, elevated or persistently high serum ferritin, and unexplained cytopenias (10). Initial manifestations of these complications may resemble the pre-existing rheumatic disease, potentially leading to delayed diagnosis and treatment. We suggest that any SS patient presenting with persistent unexplained cytopenia accompanied by the aforementioned abnormal manifestations should promptly undergo screening for hematological complications. In this instance, we promptly identified two life-threatening hematologic emergencies: iTTP and MAS. While iTTP was confirmed by severe ADAMTS13 deficiency (9), recognizing MAS required a broader approach. Although widely used, the HLH-2004 criteria were developed for pediatric HLH, limiting their sensitivity and specificity in adult autoimmune patients (10). To address this, we integrated the 2022 EULAR/ACR framework (11). Assessing the underlying SS alongside acute clinico-laboratory trajectories enabled our prompt recognition and early intervention for MAS, thereby preventing serious delays in overall management.

Despite symptom onset occurring 48 hours after initiating HCQ and TGP, the presence of severe ADAMTS13 deficiency (0%) with inhibitory antibodies confirms classic iTTP rather than drug-induced thrombotic microangiopathy (DITMA) (19). This temporal drug association is most likely coincidental, indicating underlying SS as the true pathogenic driver.

The pathogenesis of iTTP in the context of SS is primarily driven by autoantibody-mediated severe deficiency of ADAMTS13, but the underlying mechanisms linking the two conditions are multifaceted. Patients with SS, particularly those with anti-Ro/SSA antibodies, exhibit a marked upregulation of the type I interferon signature, which plays a crucial role in promoting autoreactive cell survival, activation, and autoantibody production (20, 21). Besides, SS may cause chronic endothelial damage, which increases endothelial thrombogenicity and impedes vascular endothelial restoration by releasing endothelium-derived microparticles and gradually depleting endothelial progenitor cells (22). As endothelial damage increases, von Willebrand factor is exocytosed, progressively exhausting ADAMTS13. Furthermore, literature indicates that IL-6 plays a specific and vital mechanistic role in triggering iTTP (23). While most classical pro-inflammatory factors, such as IFN-γ, are significantly decreased during acute iTTP episodes, the expression of cytokines like IL-6 and IL-10 are distinctly elevated and actively contribute to the pathology (24, 25). Studies have shown that IL-6 promotes thrombosis through a dual mechanism: the IL-6–sIL-6R complex induces a marked release of ultra-large von Willebrand factor, and IL-6 can directly inhibit the function of ADAMTS13. With increasing disease severity in iTTP, IL-6 levels show a grade-dependent increase, which was negatively correlated with a decline in ADAMTS13 activity (26). Since IL-6 is fundamentally overexpressed in the salivary glands and serum of SS patients (23), this pre-existing abundance likely fuels the thrombotic cascade. Based on these observations, we hypothesize that the pre-existing abundance of IL-6 in SS may lower the threshold for triggering the thrombotic cascade, thus serving as a potential pathogenic link to iTTP. In our presented case, because baseline IL-6 levels were unmeasured prior to iTTP onset, and current evidence shows association rather than causality, its role as a direct causative driver remains speculative and warrants further investigation.

Managing SS-associated iTTP requires aggressive intervention. Plasma exchange along with corticosteroids remain the initial treatment of iTTP, with rituximab serving as a routine immunomodulatory option (27). In our case, the patient’s condition was successfully controlled with only the basic treatment of plasma exchange and corticosteroids. Furthermore, a number of case reports have indicated that cyclosporine A and bortezomib have demonstrated significant efficacy in the treatment of refractory iTTP (28). Novel therapies, such as caplacizumab and recombinant ADAMTS13, are reshaping the therapeutic landscape (29). Given the theoretical role of IL-6 and chronic endothelial injury in driving thrombosis and autoantibody production in SS, targeting this inflammatory pathway represents a plausible therapeutic strategy. This concept is supported by a previous report in which tocilizumab successfully treated thrombotic thrombocytopenic purpura secondary to adult-onset Still’s disease (30). In our presented case, although IL-6 was measured during the acute iTTP episode, the patient’s condition was effectively controlled with conventional therapies, including plasma exchange and corticosteroids, precluding the need for IL-6-targeted biologic therapy. Furthermore, while the aforementioned single case report provides an interesting precedent, it is insufficient to establish definitive clinical efficacy. Therefore, we can only hypothesize that targeting the IL-6 pathway with agents such as tocilizumab represents a potential therapeutic avenue for refractory iTTP, a prospect that strictly warrants further systematic investigation.

Beyond these therapeutic considerations, the extreme hyperferritinemia (>40,000 ng/mL) observed during the July 2023 episode merits deeper discussion. Chart re-verification confirmed this value, ruling out transcription or assay artifacts, and comprehensive screening effectively excluded infectious triggers. This profile contrasts sharply with classical primary iTTP, where serum ferritin rarely exceeds the low thousands. Instead, such profound hyperferritinemia is a pathognomonic hallmark of a cytokine storm like HLH or MAS (11). Coupled with concurrent high fever, severe thrombocytopenia, and elevated IL-10, an incipient MAS-like pathology was likely superimposed on the acute iTTP crisis. We hypothesize that Sjögren’s-driven immune dysregulation simultaneously activated both thrombotic and macrophage-myeloid pathways, fueling this early subclinical hyperinflammation.

Notably, the acute-phase IL-6 level (9.80 pg/mL) in our patient was only modestly elevated, contrasting with the dramatic spikes (often in the hundreds) typically observed in iTTP and MAS. The absence of a dominant IL-6 surge imposes a critical constraint on attributing the clinical severity to any single pathogenic driver. Instead, this finding strongly advocates for a non-linear, multifaceted pathogenesis.

MAS is driven by impaired natural killer and CD8⁺ T cell function, causing excessive T cell activation and IFN-γ hypersecretion, triggering macrophages to release pro-inflammatory cytokines (25). Infections, particularly with Epstein-Barr virus and cytomegalovirus, are the most common triggers of MAS (31, 32). Mycoplasma-associated hemophagocytic lymphohistiocytosis is rare, with only a few documented cases (31). Through cleavage of the precursors of pro-inflammatory cytokines, Community-acquired respiratory distress syndrome toxin, an exotoxin produced by *Mycoplasma pneumoniae*, releases inflammatory cytokines including interleukin-1 beta (IL-1β), interleukin-18 (IL-18), and IFN-γ (33–35). Overproduction of IFN-γ and tumor necrosis factor-α, as well as activation of the IFN-JAK-STAT pathway play a significant role in the pathogenesis of MAS (36–38).

The mechanistic link between underlying SS and the pathogenesis of MAS likely involves distinct inflammatory networks. SS patients have high levels of type I interferons (α and β), which are primarily derived from plasmacytoid dendritic cells (20). By activating CD8⁺ T cells and immune complexes, type I interferons (α and β) induce the substantial production of inflammatory cytokines and autoantibodies, such as IFN-γ. Furthermore, the IFN-JAK-STAT pathway is also activated in patients with SS, particularly in the blood and monocytes (39). Based on these shared immunological signatures, we hypothesize that the aberrant activation of the IFN-JAK-STAT pathway and the subsequent inflammatory cytokine cascade may represent a critical mechanistic intersection, potentially predisposing patients with SS to the development of MAS.

For MAS, the combination of etoposide and dexamethasone is the first-line effective regimen (40). Additional studies are required to evaluate second- or subsequent-line options, such as anti-IFN-γ monoclonal antibodies (emapalumab), interleukin-1 receptor antagonist (anakinra) and CD52-targeted monoclonal antibody (alemtuzumab) (41). Janus Kinase (JAK) inhibitors have demonstrated significant efficacy in the treatment of MAS (38). In this presented case, despite initial therapy with pulse methylprednisolone, intravenous immunoglobulin, and etoposide, the patient maintained a high inflammatory burden. The subsequent addition of the JAK inhibitor ruxolitinib yielded a definitive therapeutic effect, leading to substantial clinical improvement and allowing for the successful tapering of glucocorticoids.

GM-CSF is a critical pro-inflammatory cytokine that directly drives macrophage phagocytosis and the secretion of pro-inflammatory cytokines through the activation of the STAT5 signaling node (42). Concurrently, GM-CSF, alongside IL-1 and tumor necrosis factor, constitutes a vicious positive feedback loop termed the “CSF network” (43). These findings suggest that GM-CSF may serve as an upstream and more specific myeloid-activating switch compared to other cytokines implicated in MAS. This hypothesis was recently corroborated by *in vitro* experiments in a study by Ding et al. (44). Furthermore, utilizing a humanized MAS preclinical model, Ding et al. demonstrated that early administration of plonmarlimab almost completely abrogated the disease cascade, highlighting its potential to prevent MAS flares in high-risk populations, such as patients with active systemic juvenile idiopathic arthritis (44). In our presented case, the patient was briefly initiated on plonmarlimab; however, the definitive therapeutic value of this drug could not be ascertained. This limitation was due to the patient’s premature voluntary withdrawal from the clinical trial, the fact that systemic inflammation was already largely controlled by that time, and the confounding residual effects of previously administered aggressive treatments, such as etoposide and ruxolitinib. Ultimately, given the multipathway nature of MAS and the relative independence of the GM-CSF axis from T-cell activation (45), combination strategies or bispecific antibodies could be effective in managing the cytokine storm while reducing the need for high-dose glucocorticoids (44).

Furthermore, the patient’s history of drug-induced liver injury constituted a crucial but non-isolated event. It could indicate a pre-existing immune dysregulation, predisposing her to a series of subsequent complications. The management of such intricate cases demands a concerted, multidisciplinary team effort (11, 46). This collaborative model was essential for navigating the intricate balance between immunosuppression and infection control, managing combination drug therapy, and addressing multi-organ involvement. This single-case report inherently lacks broad generalizability and definitive proof of precise drug causality, primarily due to the absence of longitudinal cytokine profiling and direct mechanistic assays, highlighting the need for further research. 

## Data Availability

The original contributions presented in the study are included in the article/supplementary material. Further inquiries can be directed to the corresponding author.
